# Intravitreal Co-Administration of GDNF and CNTF Confers Synergistic and Long-Lasting Protection against Injury-Induced Cell Death of Retinal Ganglion Cells in Mice †

**DOI:** 10.3390/cells9092082

**Published:** 2020-09-11

**Authors:** Simon Dulz, Mahmoud Bassal, Kai Flachsbarth, Kristoffer Riecken, Boris Fehse, Stefanie Schlichting, Susanne Bartsch, Udo Bartsch

**Affiliations:** 1 Department of Ophthalmology, Experimental Ophthalmology, University Medical Center Hamburg-Eppendorf, 20246 Hamburg, Germany; s.dulz@uke.de (S.D.); m.bassal@uke.de (M.B.); kaifla@gmx.net (K.F.); st.schlichting@uke.de (S.S.); sbartsch@uke.de (S.B.); 2 Research Department Cell and Gene Therapy, University Medical Center Hamburg-Eppendorf, 20246 Hamburg, Germany; k.riecken@uke.de (K.R.); fehse@uke.de (B.F.)

**Keywords:** axotomy, CNTF, GDNF, lentiviral vectors, neural stem cells, neuroprotection, optic nerve, regeneration, retinal ganglion cells

## Abstract

We have recently demonstrated that neural stem cell-based intravitreal co-administration of glial cell line-derived neurotrophic factor (GDNF) and ciliary neurotrophic factor (CNTF) confers profound protection to injured retinal ganglion cells (RGCs) in a mouse optic nerve crush model, resulting in the survival of ~38% RGCs two months after the nerve lesion. Here, we analyzed whether this neuroprotective effect is long-lasting and studied the impact of the pronounced RGC rescue on axonal regeneration. To this aim, we co-injected a GDNF- and a CNTF-overexpressing neural stem cell line into the vitreous cavity of adult mice one day after an optic nerve crush and determined the number of surviving RGCs 4, 6 and 8 months after the lesion. Remarkably, we found no significant decrease in the number of surviving RGCs between the successive analysis time points, indicating that the combined administration of GDNF and CNTF conferred lifelong protection to injured RGCs. While the simultaneous administration of GDNF and CNTF stimulated pronounced intraretinal axon growth when compared to retinas treated with either factor alone, numbers of regenerating axons in the distal optic nerve stumps were similar in animals co-treated with both factors and animals treated with CNTF only.

## 1. Introduction

Retinal ganglion cells (RGCs) comprise a heterogeneous group of projection neurons which integrate and transmit visual information from the retina to the brain. More than 30 distinct RGC subtypes have been identified based on morphological, physiological and molecular criteria [1,2,3] which project to more than 40 different targets in the brain [4]. In addition, RGCs display striking heterogeneity in their susceptibility to pathological conditions and their ability to regrow injured axons over long distances in response to regeneration-promoting treatments [5,6,7]. Loss of RGCs, as occurs in traumatic, inflammatory, ischemic, hereditary or glaucomatous optic neuropathies, results in visual deterioration and is a leading cause of irreversible blindness [8,9]. Treatments for these neurodegenerative disorders are either not available or have limited efficacy. Elevated intraocular pressure (IOP) is the most important risk factor for glaucoma, the most prevalent optic neuropathy. However, degeneration of RGCs progresses in a significant number of patients despite successful lowering of the IOP [10,11]. The only treatment option for late-stage optic neuropathies is a functional replacement of the lost RGCs. While cell replacement strategies have made considerable progress based on the generation of RGCs from pluripotent stem cells, restoration of relevant visual function is faced with a number of problems, including integration of appropriate RGC subtypes into the retinal circuitry, long-distance axon outgrowth from newly integrated RGCs to correct target regions and the formation of functional synaptic connections in the brain [12,13,14].

Neuroprotective approaches aim to rescue RGCs from cell death at initial stages of optic neuropathies and might be applicable to different optic neuropathies independent of the specific cause of the disease. Preclinical studies have indeed identified a number of neurotrophic factors (NTFs) that attenuate the loss of RGCs in a variety of pathological conditions [15,16,17]. Glial cell line-derived neurotrophic factor (GDNF), a member of the GDNF family of ligands [18,19], and ciliary neurotrophic factor (CNTF), a member of the neuropoietic cytokine family [20], are among these factors. Administration of either of these factors has been shown to robustly promote RGC survival in diverse animal models, including models of optic nerve injury, glaucoma or autoimmune optic neuritis [15,16]. Sustained administration of CNTF, either through adeno-associated virus (AAV)-mediated *CNTF* gene transfer or through intravitreal transplantation of CNTF-overexpressing cells, has additionally been shown to stimulate axonal regeneration of injured RGCs [21,22,23,24].

Robust promotion of RGC survival and/or axon regeneration has been observed after intravitreal transplantations of peripheral nerve grafts or intravitreal injections of diverse cell types such as Schwann cells, olfactory ensheathing cells, bone marrow-derived mesenchymal stem cells or dental pulp stem cells [25,26,27,28,29,30]. While the precise mechanism by which this neurotrophic activity is conferred to RGCs is unknown, it is generally thought to be mediated, at least in part, through the cooperative action of multiple NTFs secreted from these cells [25,31,32]. Compared to the administration of single specific factors, more pronounced, additive or even synergistic neurotrophic effects have indeed been observed in rat optic nerve injury models after administration of different NTF combinations, such as GDNF and brain-derived neurotrophic factor (BDNF) [33], BDNF and neurturin or BDNF and GDNF [34], and fibroblast growth factor-2 (FGF2), neurotrophin-3 (NT-3) and BDNF [35]. Together, the data indicate that combinatorial neuroprotective approaches represent a promising strategy to promote RGC survival under a variety of pathological conditions.

We have recently demonstrated that sustained neural stem cell-based intravitreal co-administration of GDNF and CNTF confers profound synergistic neuroprotection to injured RGCs in a mouse optic nerve crush model [36]. Co-administration of both factors resulted in the survival of ~38% RGCs two months after the nerve crush, ~4-fold more surviving RGCs than in retinas treated with either factor alone and ~14-fold more surviving RGCs than in control retinas [36]. The present study was performed to evaluate whether the synergistic neuroprotective effect of GDNF and CNTF on axotomized RGCs is long-lasting and to analyze the impact of the pronounced RGC rescue on intraretinal axon growth and axon regeneration in the optic nerve.

## 2. Materials and Methods

### 2.1. Animals

Optic nerve lesions, intravitreal cell transplantations and anterograde axonal tracing experiments were performed on adult (i.e., at least 2 months old) C57BL/6J mice. Neural stem (NS) cells were isolated from the cerebral cortices of C57BL/6J mouse embryos. All animal experiments were approved by the University and State of Hamburg Animal Care Committees (permission No. 88/15; date of approval: 08/20/2015) and were in accordance with European Union (EU) Directive 2010/63/EU.

### 2.2. Generation of GDNF- and CNTF-Expressing NS Cell Lines

**Figure 1:**
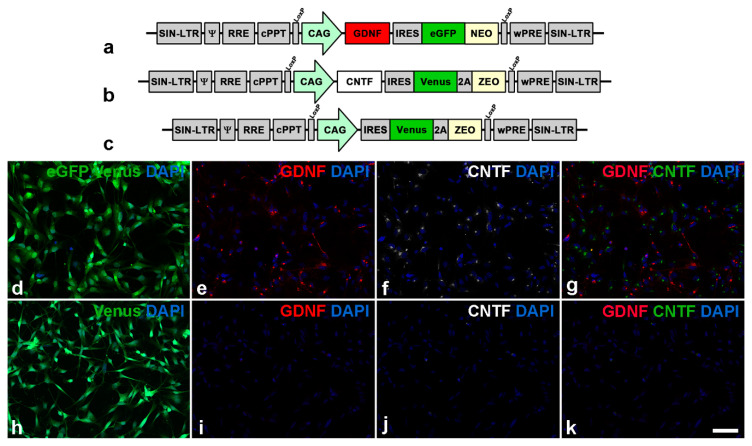
Lentiviral vectors and modified clonal neural stem cell lines. Neural stem cells were transduced with a lentiviral vector encoding glial cell line-derived neurotrophic factor (GDNF) and enhanced green fluorescent protein (eGFP) (**a**) or ciliary neurotrophic factor (CNTF) and Venus (**b**). Cells for control experiments were transduced with a vector encoding Venus only (**c**). Immunocytochemical analysis of a culture composed of a 1:1 mixture of a GDNF- and a CNTF-overexpressing neural stem cell line revealed that cells co-expressed either eGFP and GDNF (**d**,**e**) or Venus and CNTF (**d**,**f**). Cells for control experiments expressed Venus (**h**) but neither GDNF (**i**) nor CNTF (**j**). The CNTF signal in (**g**,**k**) (overlays of (**e**,**f**) and (**i**,**j**), respectively) is shown in green. Scale bar: 50 µm.

GDNF- and CNTF-expressing clonal NS cell lines were established as described based on the LeGO vector technology [23,36,37,38,39]. In brief, NS cells were transduced by spinoculation with either a bicistronic lentiviral vector encoding GDNF, together with the reporter protein enhanced green fluorescent protein (eGFP) fused to a neomycin resistance, or a bicistronic vector encoding a secretable variant of CNTF, together with the reporter protein Venus linked to zeocin resistance by a 2A peptide (Figure 1). NS cell lines with high expression levels of NTFs were established by several transductions, each followed by clonal expansion of the modified cells with the highest expression levels of the reporter proteins. A GDNF- and a CNTF-expressing clone conferring similar neuroprotective effects to axotomized RGCs were selected and used for all experiments [36]. To analyze the expression of GDNF and CNTF in undifferentiated NS cells, cultures composed of a 1:1 mixture of the GDNF- and CNTF-expressing NS cell line were fixed in 4% paraformaldehyde (PA), blocked in PBS with 0.1% bovine serum albumin and 0.3% Triton X-100 (Sigma-Aldrich, Deisenhofen, Germany) and simultaneously incubated with biotinylated polyclonal goat anti-GDNF (R&D Systems, Minneapolis, MN, USA; BAF212; 1:50) and polyclonal rabbit anti-CNTF (Santa Cruz Biotechnology, Inc., Dallas, TX, USA; sc-13996; 1:100) antibodies followed by Cy3-conjugated anti-goat and Cy5-conjugated anti-rabbit antibodies (Jackson ImmunoResearch Laboratories Inc., West Grove, PA, USA).

### 2.3. Optic Nerve Crush, Neural Stem Cell Transplantations and Anterograde Axonal Tracing

Intraorbital optic nerve lesions, criteria to include lesioned animals into the study and intravitreal NS cell transplantations are described in detail elsewhere [23,36,40,41]. In brief, animals were deeply anesthetized and optic nerves were crushed for 15 s at a distance of 0.5 to 1.0 mm from the eye using watchmakers’ forceps. One day after the crush, animals were again anesthetized, and 2µL of vitreous fluid was removed using a fine glass micropipette attached to a syringe. Subsequently, the same volume of PBS containing 7.6 × 10^5^ GDNF-, CNTF- or control-NS cells, or 7.6 × 10^5^ cells of a 1:1 mixture of GDNF- and CNTF-NS cells, was slowly injected into the vitreous cavity. Only animals that fulfilled the following criteria were included in this study: loss of the pupillary light reflex, lack of retinal bleeding and well-perfused retinal vessels and presence of a non-damaged lens. Anterograde axonal tracing experiments were performed 28 days after the nerve lesion on deeply anesthetized mice. After removal of 1.5 µL vitreous fluid, a saturated solution of biotin-N-hydroxysuccinimidester (Sigma-Aldrich) in a 1:1 dilution of dimethylformamide (Carl Roth GmbH, Karlsruhe, Germany) and ethanol was slowly injected into the vitreous cavity [23,40].

### 2.4. Analyses of Grafted Neural Stem Cells

Eight months after the intravitreal cell injections, mice were sacrificed and eyes were fixed for 1 h in 4% PA. Lenses with attached NS cells were removed and stained with goat anti-GDNF and rabbit anti-CNTF antibodies, to analyze the expression of both growth factors, or with rabbit anti-glial fibrillary acidic protein (GFAP; Dako Cytomation GmbH, Hamburg, Germany; Z0334; 1:500) and mouse anti-β-tubulin III (Sigma-Aldrich; T8660; 1:1000) antibodies, to monitor the differentiation of the grafted NS cells. Primary antibodies were detected with Cy3- and Cy5-conjugated secondary antibodies (Jackson ImmunoResearch Inc.).

### 2.5. Ganglion Cell Survival and Cell Body Size

For quantification of RGC survival, treated eyes were fixed in 4% PA for 15 min, retinas were dissected and flat-mounted onto nitrocellulose filters, fixed again for 1 h, immunostained with antibodies to brain-specific homeobox/POU protein 3A (BRN-3A; Santa Cruz Biotechnology; sc-31984; 1:200) as described [23,36] and number-coded. From each retinal quadrant (i.e., dorsal, temporal, ventral and nasal retina), 5 consecutive images were taken from the optic disc to the retina periphery, corresponding to an area of 1.9 mm^2^. RGCs were counted in these 20 images in a blinded manner using Photoshop (Adobe, San Jose, CA, USA), RGC densities were calculated, and data were analyzed with two-way ANOVA followed by Bonferroni post-hoc test using GraphPad Prism (GraphPad Software, San Diego, CA, USA). Six animals were analyzed for each experimental group and post-lesion time point.

The size of ganglion cell bodies was analyzed 8 months after the lesion in flat-mounted retinas (*n* = 5 for each experimental group) that were stained with goat anti-BRN-3A and rabbit anti-β-tubulin III (Sigma-Aldrich; T2200; 1:1000) antibodies. Confocal z-stacks through the entire thickness of the ganglion cell layer were prepared using an Olympus FluoView FV1000 confocal microscope (Olympus, Hamburg, Germany). z-projections of stacks were generated using Image J with Fiji plugin (http://imagej.nih.gov/ij/). The area of cell bodies with a clearly defined outline as assessed in the β-tubulin III immunostaining was determined in a blinded manner, and data were analyzed with one-way ANOVA followed by Bonferroni post-hoc test. Differences in the relative frequencies of different cell body size classes between experimental groups were analyzed with a chi-squared test.

### 2.6. Axonal Regeneration and Intraretinal Axon Growth

Animals with anterogradely labeled axons (*n* = 6 for each experimental group) were sacrificed one month after the optic nerve crush; eyes with attached optic nerves were immersion-fixed in 4% PA, cryoprotected and frozen. Optic nerves were longitudinally sectioned at a thickness of 25 µm, and labeled axons were visualized by incubating the sections with Cy3-conjugated streptavidin (Jackson ImmunoResearch Laboratories). Composite images of the entire nerve sections consisting of z-stacks (step size 0.3 µm) throughout the entire section thickness were prepared with an AxioObserver.Z1 microscope equipped with an ApoTome.2 (Zeiss, Oberkochen, Germany). The length of the longest regrown axon in the distal optic nerve stumps was measured in each animal using ZEN 2.1 software (Zeiss). To compare the number of regenerating axons in CNTF- and GDNF/CNTF-treated animals, we selected the three optic nerve sections from each animal that contained the longest regrown axons. All labeled axons in these sections were counted every 100 µm, starting 500 µm and ending 1700 µm distal to the lesion site. At each position, the width of the optic nerve was measured and the mean number of regenerating axons per 100 µm optic nerve width was calculated. To study intraretinal growth of RGC axons, retinas were flat-mounted 8 months after the nerve lesion and stained with polyclonal rabbit anti-β-tubulin III. Composite images consisting of z-stacks through the entire nerve fiber layer and ganglion cell layer were taken from the optic disc to the periphery of each retina using an AxioObserver.Z1 microscope equipped with ApoTome.2.

## 3. Results

### 3.1. Lentiviral Vectors and Transgene Expression in Cultured Neural Stem Cells

The generation of genetically modified NS cell lines has been described [36]. In brief, NS cells were transduced with lentiviral vectors encoding GDNF, enhanced green fluorescent protein (eGFP) and neomycin or CNTF, Venus and zeocin (ZEO) under regulatory control of a cytomegalovirus enhancer/chicken β-actin (CAG) promoter to establish GDNF-overexpressing (GDNF-NS cells) or CNTF-overexpressing (CNTF-NS cells) cell lines, respectively. NS cells for control experiments were transduced with a lentiviral vector encoding Venus and ZEO (Figure 1a–c). Clonal NS cell lines with high levels of transgene expression were established by several transductions, each followed by selection of cells with the highest expression level of the fluorescent reporter proteins and subsequent clonal expansions. Immunocytochemical analyses of cultures composed of a 1:1 mixture of GDNF- and CNTF-NS cells (GDNF/CNTF-NS cells) confirmed that all cells co-expressed either GDNF and eGFP or CNTF and Venus (Figure 1d–g). Control-NS cells expressed Venus but not GDNF or CNTF (Figure 1h–k). Analyses of culture supernatants of the same NS cell lines at similar passage numbers were performed in a previous study [36]. Experiments revealed secretion of 156.4 ± 4.8 ng GDNF per 10^5^ GDNF-NS cells in 24 h and 87.2 ± 10.1 ng CNTF per 10^5^ CNTF-NS cells in 24 h, respectively. Similar secretion levels were found in both cell lines after an additional ~20 passages, indicating stable expression of both factors. GDNF or CNTF was not detected in supernatants from control-NS cells [36].

### 3.2. Survival, Differentiation and Transgene Expression in Grafted Neural Stem Cells

**Figure 2:**
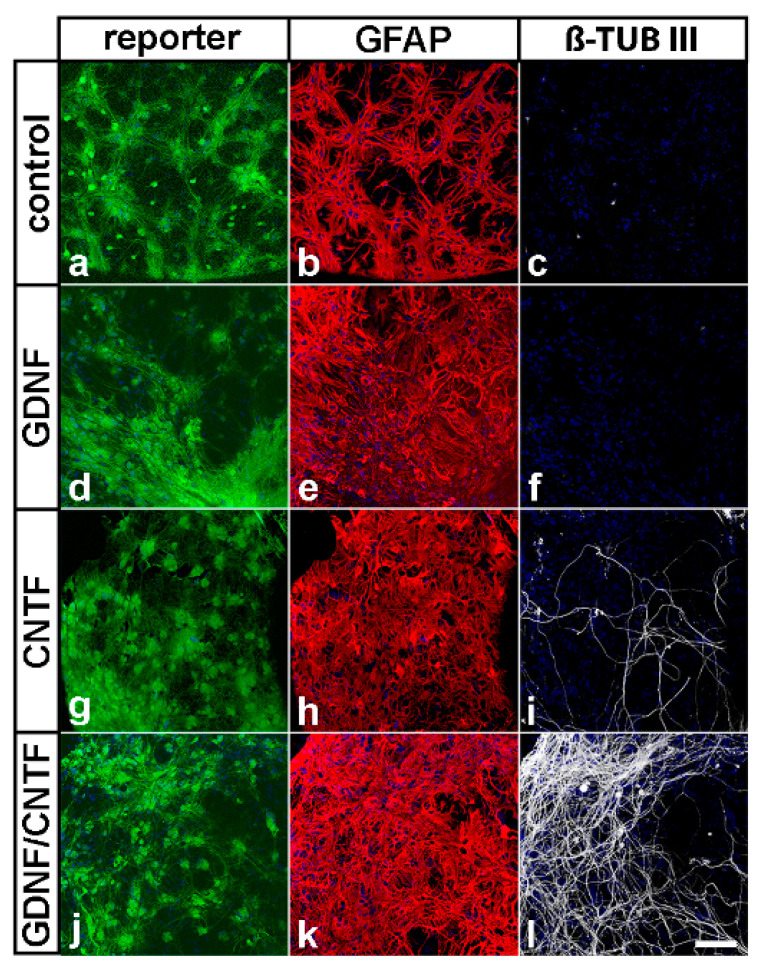
Survival and differentiation of intravitreally grafted neural stem cells. Grafted eGFP- or Venus-positive cells (**a**,**d**,**g**,**j**) were attached to the posterior surface of the lenses, where they survived for 8 months after transplantation. All NS cell lines were preferentially differentiated into GFAP-positive astrocytes (**b**,**e**,**h**,**k**). A few CNTF- (**i**) and a significant fraction of GDNF/CNTF-NS cells (**l**) were additionally differentiated into β-tubulin III-positive nerve cells. Neuronal differentiation of control-NS (**c**) or GDNF-NS cells (**f**) was not observed. Note the numerous β-tubulin III-positive neurites in animals with co-grafted GDNF- and CNTF-NS cells. β-TUB III: β-tubulin III; GFAP: glial fibrillary acidic protein. Scale bar: 100 µm.

In previous studies, we have shown that grafted GDNF-NS cells and CNTF-NS cells survive at least 2 and 4 months, respectively, in the vitreous cavity of adult mice [23,36]. To study survival, transgene expression and differentiation of grafted NS cells after an extended period of time after transplantation, donor cells were analyzed 8 months after intravitreal injection. In all experimental groups, eGFP- or Venus-positive donor cells were found to be attached to the lenses where the majority of cells were differentiated into GFAP-positive astrocytes (Figure 2). While neuronal differentiation of control- or GDNF-NS cells was not observed (Figure 2c,f), we found a few β-tubulin III-positive donor cells in eyes that received injections of CNTF-NS cells (Figure 2i). Notably, neuronal differentiation of GDNF/CNTF-NS cells was significantly more pronounced when compared to CNTF-NS cells, as indicated by a dense network of β-tubulin III-positive neurites (Figure 2l and Figure S1).

**Figure 3:**
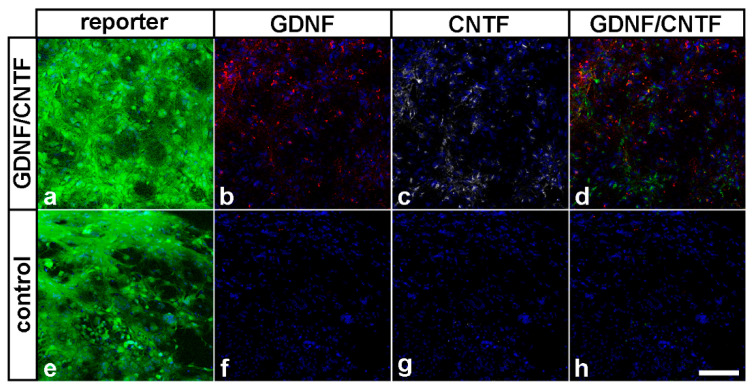
Transgene expression in grafted NS cells eight months after transplantation. Eight months after transplantation, GDNF/CNTF-NS cells expressed eGFP or Venus (**a**) together with GDNF (**b**) or CNTF (**c**). Control-NS cells expressed Venus (**e**) but not GDNF (**f**) or CNTF (**g**). The CNTF signal in (**d**) and (**h**) (overlays of (**b**,**c**) and (**f**,**g**), respectively) is shown in green. Scale bar: 100 µm.

Importantly, we found robust expression of GDNF, CNTF and the fluorescent reporter proteins in donor cells derived from GDNF- or CNTF-NS cells 8 months after cell transplantation (Figure 3a–d). Donor cells derived from control-NS cells expressed the reporter protein Venus but neither GDNF nor CNTF (Figure 3e–h). Adverse effects of the grafted cells on the morphology of host eyes or formation of tumors was not observed in any of the experimental animals.

### 3.3. Long-Term Survival of Axotomized Ganglion Cells

**Figure 4:**
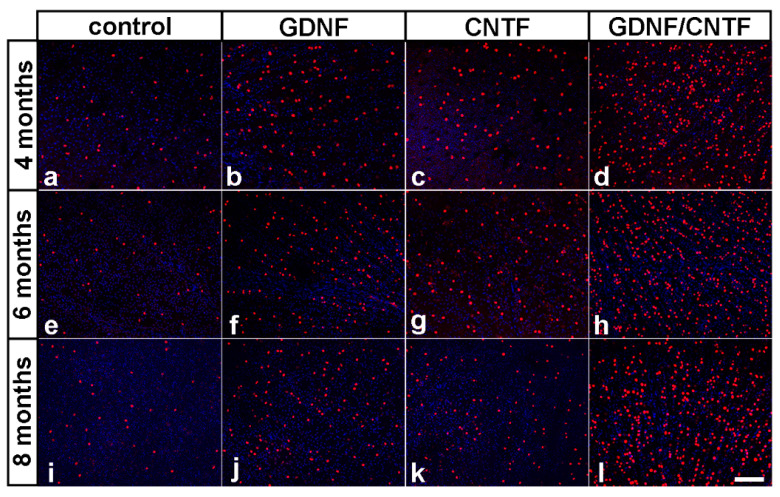
Long-term survival of axotomized ganglion cells in GDNF-, CNTF- and GDNF/CNTF-treated retinas. BRN-3A-positive ganglion cells in flat-mounted retinas 4 (**a**–**d**), 6 (**e**–**h**) and 8 (**i**–**l**) months after an optic nerve lesion and intravitreal transplantations of control-NS (**a,e,i**), GDNF-NS (**b**,**f**,**j**), CNTF-NS (**c**,**g**,**k**) or GDNF/CNTF-NS cells (**d**,**h**,**l**). GDNF- or CNTF-treated retinas contained significantly more ganglion cells than control retinas at all post-lesion time points. Note the markedly increased density of surviving ganglion cells in retinas co-treated with GDNF and CNTF when compared with retinas treated with either factor alone. All images were taken close to the optic disc. Scale bar: 50 µm.

To analyze whether the intravitreal administration of GDNF and CNTF conferred long-term protection to the axotomized RGCs, surviving ganglion cells were visualized in retinal flat-mounts using anti-BRN-3A antibodies. Only a few BRN-3A-positive RGCs were present in eyes with injected control-NS cells 4, 6 and 8 months after the lesion (Figure 4a,e,i). In GDNF- (Figure 4b,f,j) and CNTF-treated retinas (Figure 4c,g,k), in comparison, the number of surviving RGCs was significantly increased to a similar extent when compared to control retinas at all post-lesion time points. Remarkably, the density of BRN-3A-positive RGCs was markedly increased in retinas simultaneously treated with GDNF and CNTF (Figure 4d,h,l) when compared to retinas treated with either factor alone. Furthermore, qualitative inspection of flat-mounted retinas suggested similar RGC densities in each experimental group at the different post-lesion time points (Figure 4). A BRN-3A-stained retinal flat-mount from a healthy adult animal with an uninjured optic nerve is shown in Figure S2 for comparison.

**Figure 5:**
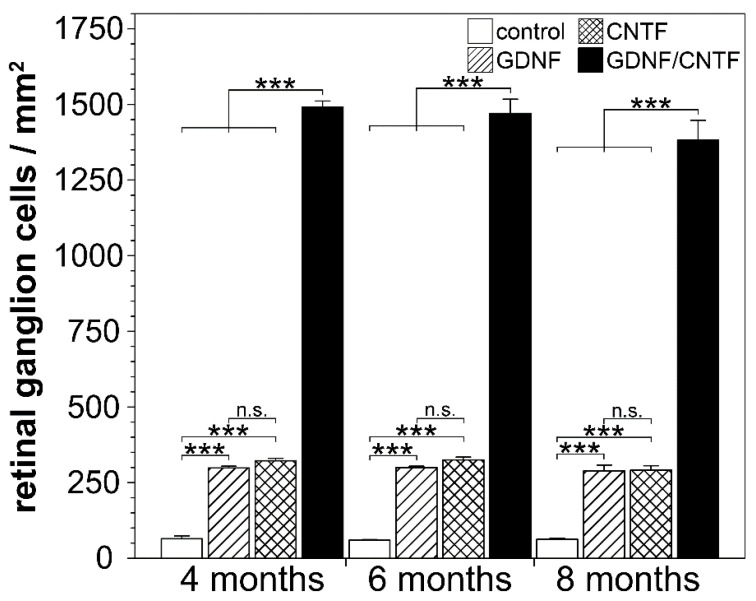
Quantitative analysis of retinal ganglion cell survival. The density of BRN-3A-positive ganglion cells was determined in flat-mounted retinas 4, 6 and 8 months after an optic nerve crush and intravitreal transplantations of control-NS (open bars), GDNF-NS (hatched bars), CNTF-NS (cross-hatched bars) or GDNF/CNTF-NS cells (filled bars). The density of retinal ganglion cells in GDNF- or CNTF-treated retinas was significantly higher than in control retinas at all post-lesion time points. Note that the number of surviving ganglion cells was markedly increased in retinas simultaneously treated with GDNF and CNTF when compared to retinas treated with either GDNF or CNTF. Note also the similar density of ganglion cells in each experimental group at the different post-lesion time points. Each bar represents the mean number (±SEM) of retinal ganglion cells per mm^2^ from six retinas. n.s.: not significant; ***: *p* < 0.001 according to two-way ANOVA followed by Bonferroni post-hoc test.

Determination of RGC densities (*n* = 6 for each experimental group) revealed 64.5 ± 9.2 (mean ± SEM), 59.4 ± 2.8 and 62.6 ± 3.7 RGCs per mm^2^ in control retinas 4, 6 and 8 months after the lesion, respectively. Retinas treated with either GDNF or CNTF contained similar numbers of surviving RGCs that were significantly higher than in control retinas at all post-lesion time points (GDNF: 298.7 ± 5.6, 299.8 ± 4.7 and 289.2 ± 18.8 RGCs/mm^2^ 4, 6 and 8 months post-lesion, respectively; CNTF: 322.2 ± 8.0, 325.3 ± 9.4 and 291.3 ± 14.2 RGCs/mm^2^ 4, 6 and 8 months post-lesion, respectively). The combined treatment with GDNF and CNTF had a profound synergistic rescue effect on axotomized RGCs, with 1491.2 ± 20.0, 1470.1 ± 47.1 and 1382.8 ± 64.6 RGCs/mm^2^ 4, 6 and 8 months after the lesion, respectively (Figure 5). GDNF/CNTF-treated retinas thus contained ~4.7-fold more RGCs than GDNF- or CNTF-treated retinas and ~22-fold more RGCs than control retinas 8 months after the lesion. Notably, there was no significant loss of RGCs in GDNF-, CNTF- or GDNF/CNTF-treated retinas between consecutive analysis time points (two-way ANOVA followed by Bonferroni post-hoc test). In a previous study, we had analyzed the impact of the same clonal NS cell lines on RGC survival 0.5, 1 and 2 months after an intraorbital optic nerve crush [36]. When data from that study were included in the analysis (Figure S3), there was no significant loss of RGCs in GDNF-, CNTF- and GDNF/CNTF-treated retinas between consecutive analysis time points beginning with 1 month after the lesion. Furthermore, we detected no significant decrease in the number of RGCs in GDNF- or CNTF-treated retinas between the second and eighth month after the lesion and only a moderate (~8.2%) but significant (*p* < 0.05) loss during the same time period in GDNF/CNTF-treated retinas.

### 3.4. Axon Regeneration into the Distal Optic Nerve Stumps

**Figure 6:**
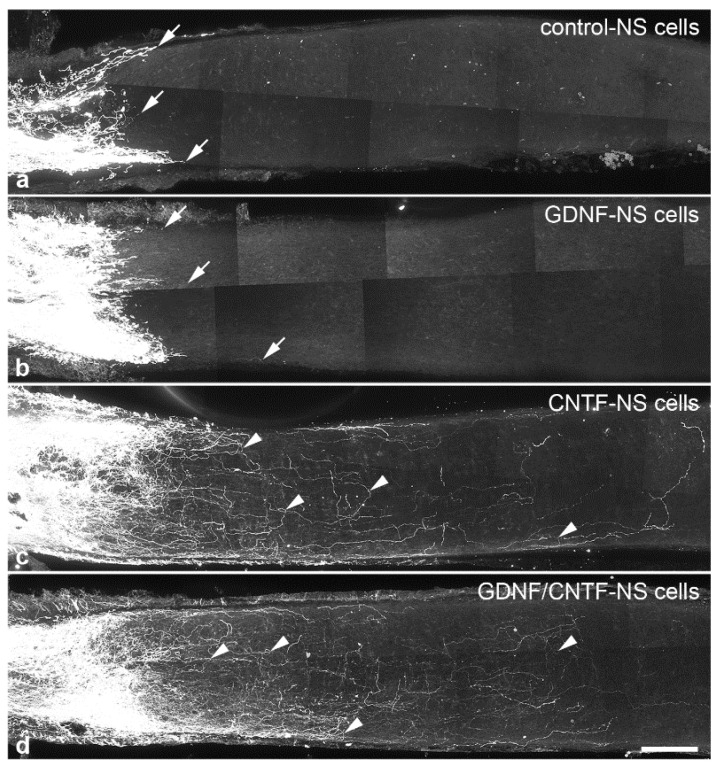
Regeneration of intraorbitally lesioned ganglion cell axons into the distal optic nerve stump. RGC axons were anterogradely labeled 1 month after an optic nerve crush in animals with grafted control- (**a**), GDNF- (**b**), CNTF- (**c**) or GDNF/CNTF- (**d**) NS cells. In mice with grafted control- or GDNF-NS cells, only a few axons regrew for only a short distance into the distal nerve stump (white arrows in (**a**) and (**b**) label the tips of some regrown axons). In animals with grafted CNTF- and GDNF/CNTF-NS cells, in comparison, significantly more RGC axons were regrown across the lesion site and extended for significantly longer distances into the distal nerve stumps. Axons in CNTF- and GDNF/CNTF-treated mice followed irregular trajectories and some made U-turns (arrowheads in (**c**,**d**)) and grew back towards the retina. Scale bar: 100 µm.

**Figure 7:**
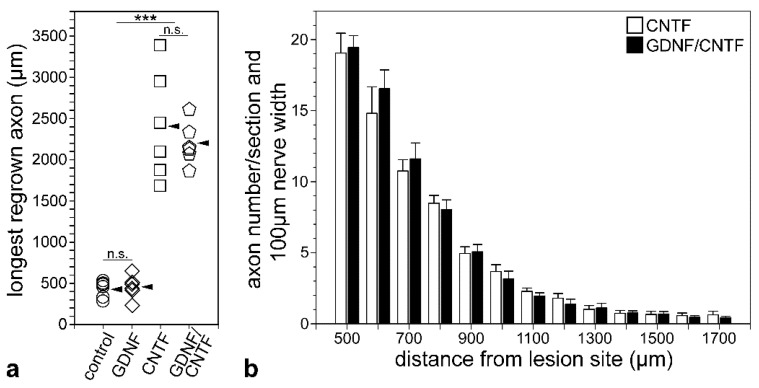
Quantitative analyses of axon regeneration. The length of the longest regrown axon was determined in the distal optic nerve stumps of mice with grafted control- (circles), GDNF- (diamonds), CNTF- (squares) and GDNF/CNTF- (pentagons) NS cells (**a**). While axons in control and GDNF-treated animals extended for only around 500 µm into the distal nerve stumps, axons in CNTF- and GDNF/CNTF-treated animals extended for up to 3400 and 2600 µm, respectively, distal to the lesion site. Arrowheads indicate mean values (*n* = 6 for each experimental group). The number of regrown axons in optic nerve sections from CNTF- (open bars) and GDNF/CNTF- (filled bars) treated animals at different positions distal to the lesion site (**b**). Note the similar number of regenerating axons in both experimental groups at all positions analyzed. Each bar represents the mean number (±SEM) of axons per 100 µm nerve width in 25 µm thick nerve sections from 6 animals. n.s.: not significant; ***: *p* < 0.001 according to one-way ANOVA followed by Bonferroni post-hoc test.

To study the impact of the markedly increased number of surviving RGCs in GDNF/CNTF-treated retinas on axonal regeneration, we determined the length of the longest regrown axon and the number of regrowing axons in the distal optic nerve stumps 1 month after the lesion. In control and GDNF-treated animals, only a few axons extended for short distances into the distal nerve stumps (Figure 6a,b). The length of the longest regrown axon in control and GDNF-treated mice was 434.3 ± 41.1 µm (mean ± SEM) and 460.2 ± 56.8 µm, respectively (*n* = 6 for each experimental group; not significantly different according to the one-way ANOVA followed by Bonferroni post-hoc test (*p* > 0.05); Figure 7a). Length of the longest regrown axons in CNTF- (2409.8 ± 268.5 µm) and GDNF/CNTF-treated (2204.5 ± 103.3 µm) animals was significantly increased to a similar extent (*p* > 0.05) when compared to control and GDNF-treated mice (*p* < 0.001 for all comparisons; Figure 7a).

Their irregular trajectory indicates that they represent regenerated axons rather than axons that were spared by the nerve lesion. In fact, several axons in both experimental groups made U-turns and grew back towards the retina (Figure 6c,d). The length of the longest regrown axon in mice with grafted CNTF-NS cells or GDNF/CNTF-NS cells was similar, with 2409.8 ± 268.5 µm in CNTF-treated and 2204.5 ± 103.3 µm in GDNF/CNTF-treated animals (*n* = 6 for each experimental group; Figure 7a). Next, we analyzed the impact of the increased number of surviving RGCs in GDNF/CNTF-treated retinas on the number of regrowing axons. Axons were counted in 25 µm thick sections every 100 µm, beginning 500 and ending 1700 µm distal to the lesion site, and numbers were normalized to 100 µm nerve width. Data revealed no significant difference in the number of regenerating axons between CNTF- and GDNF/CNTF-treated animals at any position analyzed (mixed two-way ANOVA followed by Bonferroni post-hoc test; Figure 7b).

### 3.5. Intraretinal Growth of Ganglion Cell Axons

**Figure 8:**
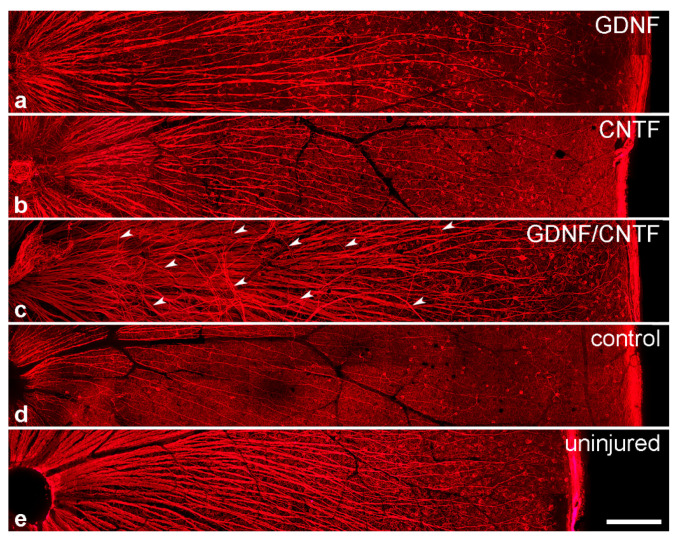
Intraretinal growth of retinal ganglion cell axons. β-tubulin III-positive ganglion cell axons in flat-mounted retinas 8 months after an intraorbital crush and intravitreal transplantations of GDNF- (**a**), CNTF- (**b**), GDNF/CNTF- (**c**) or control- (**d**) NS cells. A retina from an adult animal with an uninjured optic nerve is shown for comparison (**e**). Axon fascicles in GDNF-, CNTF- and GDNF/CNTF-treated retinas (**a**–**c**) were significantly thicker than in control retinas (**d**). Note the aberrant trajectories of some axon fascicles (some labeled with white arrowheads in (**c**)) in animals with grafted GDNF/CNTF-NS cells. Scale bar: 200 µm.

Intraretinal growth of ganglion cell axons was studied in flat-mounted retinas 8 months after the nerve lesion and cell transplantation. In retinas from healthy adult animals with uninjured optic nerves, axon fascicles follow a straight course towards the optic disc in a radial pattern (Figure 8e). A similar organization of axon fascicles was observed in mice with lesioned optic nerves and grafted control-NS cells, with the only exception that fascicles were significantly thinner, as expected as a result of the pronounced injury-induced RGC loss (Figure 8d). Axon fascicles in GDNF- and CNTF-treated retinas, in comparison, were significantly thicker than in mice with grafted control cells, in line with the higher number of RGCs that survived the nerve lesion (Figure 8a,b). A few axon fascicles in GDNF- and CNTF-treated retinas took an aberrant course. Such fascicles were mainly observed in close proximity to the optic disc (Figure S4). In GDNF/CNTF-treated animals, axon fascicles were markedly thicker than in all other experimental groups, reflecting the higher number of rescued RGCs (Figure 8c). In addition, axon fascicles were markedly more disorganized than in GDNF- or CNTF-treated retinas, with more fascicles that followed irregular trajectories and some crossing adjacent fascicles (Figure 8c and Figure S4).

### 3.6. Soma Size of Ganglion Cells

The soma size of RGCs 8 months after the lesion was not significantly different between CNTF-treated retinas, GDNF-treated retinas, control retinas and retinas from uninjured animals (Figure S5A). However, the size of RGC cell bodies was significantly increased in eyes with grafted GDNF/CNTF-NS cells (120.3 ± 10.1 µm^2^; mean ± SEM) when compared to eyes with grafted control-NS cells (91.4 ± 4.3 µm^2^) or eyes with uninjured nerves (86.7 ± 2.4 µm^2^; *p* < 0.05, one-way ANOVA followed by Bonferroni post-hoc test). The observed increase in soma size in GDNF/CNTF-treated retinas was the result of a significantly increased number of RGCs with a soma size of 200–400 µm^2^ when compared to all other experimental groups (*p* < 0.05, chi-squared test; Figure S5B). Some ganglion cells in GDNF/CNTF-treated retinas had a soma size of 400–600 µm^2^. RGCs with cell bodies of this size were not detected in any other experimental group or in retinas from uninjured mice (Figure S5B).

## 4. Discussion

We have recently shown that neural stem cell-based intravitreal administration of either GDNF or CNTF protects a small but significant number of RGCs against injury-induced cell death for up to two months after an intraorbital optic nerve crush [36], in line with the well documented ability of each factor to rescue RGCs under a variety of pathological conditions [15,16,17]. More importantly, we have demonstrated that co-administration of both NTFs conferred pronounced synergistic neuroprotective effects to injured RGCs, with ~38% of all RGCs still being viable two months after the lesion, ~4-fold more RGCs than in retinas treated with either factor alone [36]. Here, we analyzed whether the synergistic neuroprotective effect of GDNF and CNTF is long-lasting and studied the impact of this combinatorial neuroprotective approach on RGC soma size, intraretinal axon growth and axonal regrowth into the distal optic nerve stumps.

Analyses of experimental eyes 8 months after the nerve lesion and cell transplantation revealed the presence of numerous donor cells that had formed dense cell layers on the posterior poles of the lenses and co-expressed either eGFP and GDNF or Venus and CNTF. Qualitative inspection revealed no obvious differences with regard to size and cell density between donor cell layers found in the present study and donor cell layers found in previous short-term experiments (i.e., 7 days to 2 months after transplantation [36,37,42]), indicating long-term survival of grafted cells. Adverse effects of the transplanted cells on the morphology of host eyes or formation of tumors were not observed in any experimental animal, in agreement with the observation that NS cells rapidly cease proliferation after intravitreal transplantation [37]. While all control- and GDNF-NS cells were differentiated into astrocytes, a few CNTF-NS cells were additionally differentiated into neurons, in line with our previous studies [23,36,37,42]. In eyes co-treated with GDNF and CNTF, in comparison, a significant fraction of donor cells was identified as β-tubulin III-positive neurons, which had elaborated a dense network of neurites. NS cells cultivated under the conditions used in the present study represent a homogeneous population of tripotent neural stem cells that display high neurogenic differentiation potential [43,44,45]. While we cannot exclude the notion that the combination of GDNF and CNTF promoted neuronal differentiation of the grafted NS cells, we did not observe obvious differences between the number of nerve cells in differentiated cultures composed of a mixture of GDNF- and CNTF-NS cells and cultures composed of GDNF-, CNTF- or control-NS cells only. We thus consider it more likely that a significant fraction of NS cells had differentiated into neurons directly after transplantation and that these neurons were then rescued from cell death by both factors, but not by either factor alone, during the 8 months post-transplantation period.

Long-term survival and stable transgene expression of donor cells correlated with a long-lasting rescue of some axotomized RGCs in retinas treated with GDNF or CNTF. Eight months after the lesion, GDNF- or CNTF-treated retinas contained around 300 RGC/mm^2^, ~4.6-fold more than control retinas. Using the same method and the same RGC marker for quantification as in the current study, we found 3993.8 ± 54.4 (mean ±SEM) BRN3A-positive RGCs/mm^2^ in retinas from adult healthy mice with uninjured optic nerves [23]. Thus, each factor alone conferred long-term protection to only a minor RGC population, accounting for ~7.5% of all BRN3A-positive RGCs. Notably, however, there was no significant loss of RGCs between the fourth and eighth post-lesion month, neither in GDNF- nor in CNTF-treated retinas. More importantly, numbers of surviving RGCs were markedly higher in retinas co-treated with GDNF and CNTF, with ~35% of RGCs still being viable eight months after the nerve crush, ~5-fold more than in retinas treated with either NTF. Furthermore, and similar to GDNF- or CNTF-treated retinas, there was no significant loss of RGCs between successive analysis time points (i.e., 4, 6 and 8 months post-lesion), and only a moderate albeit significant loss of ~7.3% between the fourth and eighth post-lesion month. Considering in addition the survival rates of RGCs in mice treated with the same clonal NS cell lines 0.5, 1 and 2 months after the lesion reported in a previous study [36], there was no significant RGC loss in GDNF-, CNTF and GDNF/CNTF-treated retinas between successive analysis time points beginning from the first month after the nerve crush and only a moderate loss of ~8.2% between the second and eighth post-lesion months in GDNF/CNTF-treated retinas. We also found that the average size of ganglion cell bodies in GDNF/CNTF-treated retinas was significantly increased when compared with control retinas or retinas from uninjured animals, indicative of either a preferential rescue of large-sized RGCs or stimulation of RGC growth. A population of abnormally large ganglion cell bodies present only in GDNF/CNTF-treated retinas but not in retinas of all other experimental groups or in retinas from uninjured mice supports the latter possibility. The phosphatidylinositol 3′-kinase (PI3K)/protein kinase B (AKT)/mechanistic target of the rapamycin (mTOR) signaling pathway is involved in the control of cell size [46,47], and expression of a constitutively active variant of the downstream effector ribosomal protein S6 kinase 1 (S6K1) in RGCs markedly increased cell size in a mouse optic nerve crush model [48]. It is thus tempting to speculate that, at least to some extent, the increased size of RGCs in GDNF/CNTF-treated retinas is the result of a concerted activation, directly and indirectly through the induction of Müller cells to secrete additional growth factors such as osteopontin (OPN) [49,50], of this signaling cascade. Together, our results indicate that the co-administration of GDNF and CNTF confers not only synergistic but possibly lifelong protection against axotomy-induced cell death.

Most studies have quantified the survival of RGCs already a few weeks after optic nerve injury and neuroprotective treatment. For example, AAV-mediated *GDNF* gene transfer after transection of the optic nerve in rats resulted in the survival of 1.6- to 1.8-fold more RGCs than in control retinas 14 days after the lesion [51,52]. With our NS-cell-based delivery approach, in comparison, we found 2.4-fold more viable RGCs in GDNF-treated retinas than in control retinas at this early post-lesion time point [36]. Furthermore, retinas with AAV-mediated overexpression of CNTF contained 3.9- and 1.5-fold more viable RGCs than controls 7 [21] and 8 weeks [24] after an optic nerve lesion, respectively. In comparison, we found 3.7-fold more surviving RGCs in eyes with grafted CNTF-NS cells than in eyes with grafted control-NS cells 8 weeks after the lesion [36]. These data indicate that the rescue effects achieved with our cell-based approach are in the range of those achieved with AAV-mediated NTF gene transfer. Finally, when an AAV-CNTF treatment was combined with an autologous and “blind-ended” peripheral nerve graft sutured to the proximal optic nerve stump, the fraction of surviving RGCs 7 weeks after optic nerve injury increased from ~17% to ~25% [21]. Notably, the number of viable RGCs remained relatively stable for up to 15 months after the lesion [53], similar to the robust and sustained promotion of RGC survival observed after co-administration of GDNF and CNTF. A long-lasting rescue through neuroprotective treatments has been reported also for other retinal cell types. For instance, continuous delivery of CNTF to the retina of rhodopsin-deficient mice conferred lifelong protection to a significant fraction of cone photoreceptors [54] and prevented the loss of cones over a time period of up to ~ 3 years in patients with hereditary retinal dystrophies [55].

Even though it is unknown how the synergistic rescue effect of GDNF and CNTF is mediated, it is likely the result of a combination of direct neuroprotective activities of both NTFs through binding to their cognate receptors expressed by RGCs [34,56,57,58,59] and indirect neuroprotective activities. For instance, GDNF and CNTF have both been shown to induce Müller cells to secrete additional neuroprotective factors known to promote RGC survival, such as BDNF, FGF-2, leukemia inhibitory factor and OPN [49,60,61,62]. In addition, both NTFs have been demonstrated to upregulate sodium-dependent glutamate/aspartate transporter 1 (GLAST-1) in retinal glia cells, thereby enhancing the uptake of extracellular glutamate and attenuating glutamate-related excitotoxicity [63,64]. A critical contribution of indirect effects to the synergistic neuroprotective activity is suggested by the finding that ~35% of all RGCs are still viable in GDNF/CNTF-treated retinas 8 months after the lesion, but only 13%–14% of all RGCs express the GDNF receptors GDNF-family receptor-α1 (GFRα1) and the receptor tyrosine kinase rearranged during transfection (RET) [59].

Results of the present and a previous study [36] confirm and extend the view that sustained co-administration of different NTFs provides a promising strategy to effectively promote the survival of RGCs under pathological conditions. For instance, intravitreal co-injections of recombinant GDNF and BDNF into a rat optic nerve transection model resulted in the survival of more RGCs than injections of either NTF alone, although in a less than additive manner [33]. More surviving RGCs were also observed after intravitreal co-injections of GDNF and neurturin when compared to separate injections of each factor, although the rescue effect of both NTFs was again less than additive [34]. Co-injections of GDNF and BDNF or neurturin and BDNF, in comparison, rescued axotomized RGCs in an additive manner [34], and intravitreal co-transplantations of fibroblasts overexpressing FGF-2, NT-3 or BDNF into a rat optic nerve transection model promoted RGC survival and axon regeneration in a synergistic manner [35].

To analyze the impact of the markedly increased number of surviving RGCs in GDNF/CNTF-treated animals on axon regeneration, we determined the length and number of regrowing RGC axons in the distal optic nerve stumps 1 month after the lesion. In control mice, only a few axons grew for only short distances across the lesion site into the distal nerve stumps, as expected. Similar results were obtained for GDNF-treated animals, in line with a previous study demonstrating that GDNF promotes RGC survival but not axon regrowth [65]. In CNTF-treated animals, in comparison, some axons regrew over long distances into the distal nerve stumps, with a few extending for more than 2 mm distal to the lesion site. Axons followed tortuous paths and some made U-turns and grew back towards the eye. A detailed analysis of the circuitous growth pattern has been performed in studies in which axon regeneration was stimulated through a conditional co-deletion of phosphatase and tensin homolog (PTEN) and suppressor of cytokine signaling 3 (SOCS3) in RGCs combined with intravitreal CNTF injections or through AAV-mediated *CNTF* gene transfer to retinal glia cells [24,66]. These studies revealed that regenerating axons formed branches and ~40% made at least one U-turn, with some extending back towards the retina. These tortuous trajectories of regenerating axons, together with pathfinding errors at the optic chiasm and in the brain, observed in experiments that have achieved long-distance regrowth through combinatorial regeneration-promoting treatments, are among the obstacles that limit meaningful functional recovery after injury [5,67,68].

One month after the crush, GDNF/CNTF-treated retinas contained ~1,570 RGCs/mm^2^ as opposed to only ~480 viable RGCs/mm^2^ in CNTF-treated retinas [36]. Despite the 3.3-fold higher number of surviving RGCs in the former group, the number and length of regenerating axons were similar in GDNF/CNTF- and CNTF-treated animals. A striking discrepancy between the number of viable RGCs and the number of regenerating axons in the distal optic nerve stumps is a general observation after survival- and/or regeneration-promoting treatments [5,17,68,69]. These findings suggest that different RGC subclasses require different stimuli to regenerate their axons, as has been demonstrated for αRGCs that comprise ~6% of all RGCs and regenerate their axons upon OPN overexpression and administration of insulin-like growth factor 1 or BDNF [70]. Combinations of different regeneration-stimulating treatments indeed resulted in markedly improved axon regeneration, eventually with a few axons reaching visual targets in the brain and restoring some visual function [5,68,71,72].

We also analyzed the organization of RGC axons in the retinal nerve fiber layer. In retinas from healthy adult mice with uninjured optic nerves, RGC axons are organized in distinct fascicles that extend in a radial pattern towards the optic disc to form the optic nerve. Eight months after the lesion, calibers of axon fascicles were markedly reduced in control retinas, moderately reduced in GDNF- and CNTF-treated retinas and slightly reduced in GDNF/CNTF-treated retinas when compared to retinas from uninjured mice, reflecting the varying extent of RGC loss in the different experimental groups. In addition, a few axons in GDNF-treated retinas followed aberrant trajectories near the optic disc. Aberrant axon growth near the optic disc was more pronounced in CNTF-treated than in GDNF-treated retinas and extensive in GDNF/CNTF-treated retinas. Thus, the co-administration of GDNF and CNTF not only resulted in a markedly increased number of surviving RGCs, but it also stimulated extensive intraretinal axon growth without, however, improving axon regeneration into the optic nerve when compared to CNTF-treated animals. Similar to these findings, repeated intravitreal injections of BDNF stimulated profuse axon growth around the optic disc in a rat optic nerve transection model but did not increase the number of regenerating axons into a peripheral nerve graft [73]. Aberrant intraretinal axonal growth preferentially near the optic disc has also been observed after optic nerve injury and intravitreal transplantations of Schwann cells [74] or AAV-mediated expression of CNTF in Müller cells [24]. The aberrantly growing axons might represent collaterals originating from intraretinally located RGC axons. However, as discussed above, a significant fraction of regenerating axons in mice with a PTEN/SOCS3 co-deletion and intravitreal CNTF injections or AAV-mediated CNTF expression in retinal glial cells made U-turns in the distal nerve stumps and grew back towards the retina [24,66]. In addition, a recent study showed that axons, presumably originating from αRGCs, regrew over considerable distances within the proximal nerve stumps, where they followed tortuous trajectories and formed branches even in the absence of any regeneration-promoting treatment. Treatment with CNTF aggravated the circuitous axon growth in the proximal nerve stumps [75]. It is thus possible that at least a fraction of the aberrantly growing axons near the optic disc correspond to misdirected axons that regrew from the optic nerve back into the retina. In any case, it remains to be seen whether intraretinal axon growth is a common phenomenon of neuroprotective and regeneration-promoting treatments and whether there is a link between intraretinal axon growth and sustained RGC survival, e.g., through the formation of ectopic synapses.

Taken together, our data demonstrate that intravitreal transplantations of lentivirally modified NS cells represent an efficient means to deliver secreted gene products over an extended period of time to the murine retina. Clinical trials have demonstrated the principal feasibility to translate cell-based neuroprotective approaches into clinical applications using the so-called encapsulated cell technology. This technology employs intravitreal implantations of small semipermeable devices that are placed outside the visual axis and contain CNTF-overexpressing cells to continuously administer the cytokine to the retina. The implants have a calculated half-life of more than 4 years, as judged from CNTF levels in the vitreous [76], and their ability to attenuate retinal degeneration is being tested in patients with diverse retinal disorders [55,77,78], including glaucoma (www.clinicaltrials.gov, NCT02862938).

In summary, we have demonstrated that GDNF and CNTF synergistically protect retinal ganglion cells against injury-induced cell death over an extended period of time in a mouse optic nerve crush model. Results confirm and extend the view that combinatorial neuroprotective treatments represent a promising strategy to promote the survival of RGCs under pathological conditions. It will be interesting to evaluate the impact of combined administration of both NTFs on RGC survival in animal models of milder and more slowly progressing non-traumatic optic neuropathies, such as glaucoma.

## Supplementary Materials

The following are available online at https://www.mdpi.com/2073-4409/9/9/2082/s1, Figure S1: Differentiation of intravitreally grafted GDNF/CNTF-NS cells, Figure S2: Representative images of BRN-3A-stained retinal flat-mounts, Figure S3: Quantitative analysis of retinal ganglion cell survival, Figure S4: Intraretinal growth of retinal ganglion cell axons; Figure S5: Soma size of retinal ganglion cells.
