# Supplementary material for: Intravitreal Co-Administration of GDNF and CNTF Confers Synergistic and Long-Lasting Protection against Injury-Induced Cell Death of Retinal Ganglion Cells in Mice
Source: Cells. 2020 Sep 11;9(9):2082. doi: 10.3390/cells9092082 (PMC7565883; doi:10.3390/cells9092082)
Supplement: Supplementary file 1 [file cells-09-02082-s001.pdf]

## Supplementary Materials

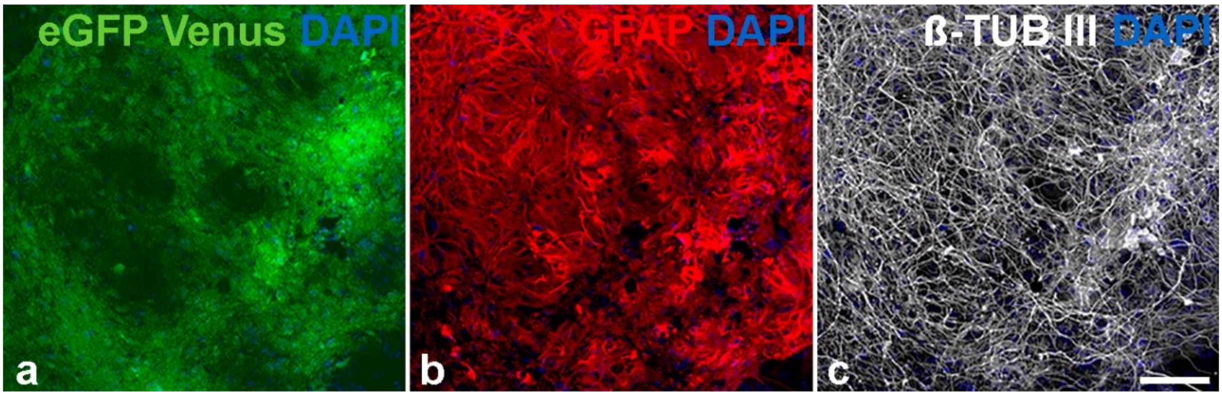


**Figure S1.** Differentiation of intravitreally grafted GDNF/CNTF-NS cells. Eight months after intravitreal transplantation, GDNF/CNTF-NS-cells were identified by their expression of the reporter protein eGFP or Venus (**a**). Grafted cells were differentiated into GFAP-positive astrocytes (**b**) or ß tubulin III positive neurons (**c**). Note the high density of ß tubulin III-positive neurites (**c**). ß-TUB III: ß-tubulin III; DAPI: 4′,6-diamidino-2-phenylindole; eGFP: enhanced green fluorescent protein; GFAP: glial fibrillary acidic protein. Scale bar: 100 µm.

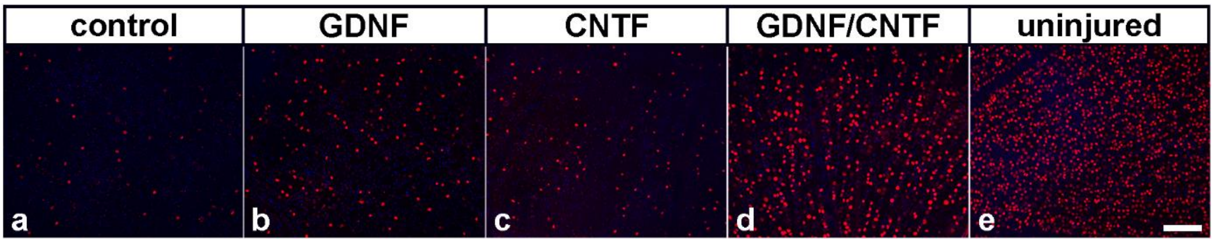


**Figure S2.** Representative images of BRN-3A-stained retinal flatmounts. Images were taken from animals 8 months after an optic nerve lesion and intravitreal transplantation of control-NS (a), GDNF-NS (b), CNTF-NS (c) or GDNF/CNTF-NS cells (d). A retinal flatmount from an adult mouse with an uninjured optic nerve is shown in (e) for comparison. All images were taken close to the optic disc. Scale bar: 50 μm.

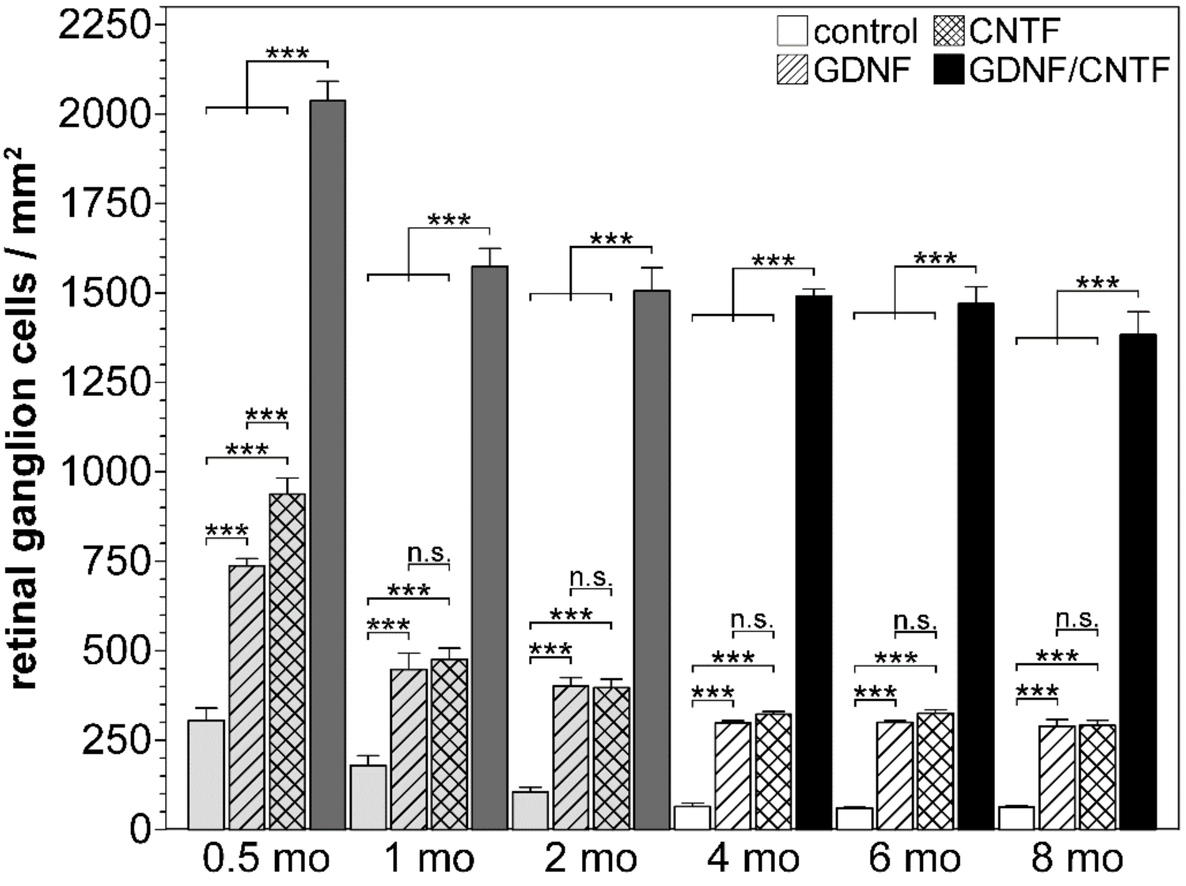


**Figure S3.** Quantitative analysis of retinal ganglion cell survival. The density of BRN-3A-positive retinal ganglion cells in animals with intravitreally grafted control-NS (open bars), GDNF-NS (hatched bars), CNTF-NS (cross-hatched bars) and GDNF/CNTF-NS cells (filled bars) 0.5, 1, 2, 4, 6 and 8 months after an optic nerve crush and cell transplantation. The number of ganglion cells in GDNF- or CNTF-treated eyes was significantly higher than in control eyes at all post-lesion time points. Note the markedly increased density of ganglion cells in eyes with grafted GDNF/CNTF-NS cells when compared to eyes that received injections of either GDNF-NS or CNTF-NS cells. Note also the similar density of ganglion cells at successive analysis time points in GDNF-, CNTF- and GDNF/CNTF-treated retinas beginning from the first month after the lesion. Each bar represents the mean number of ganglion cells per $mm^2$ (±SEM) from six retinas. Bars for the 0.5, 1 and 2 months post-lesion time points (gray bars) were reproduced with permission from a previous study [36]. n.s.: not significant; ***, $p<0.001$ according to the two-way ANOVA followed by a Bonferroni post-hoc test.

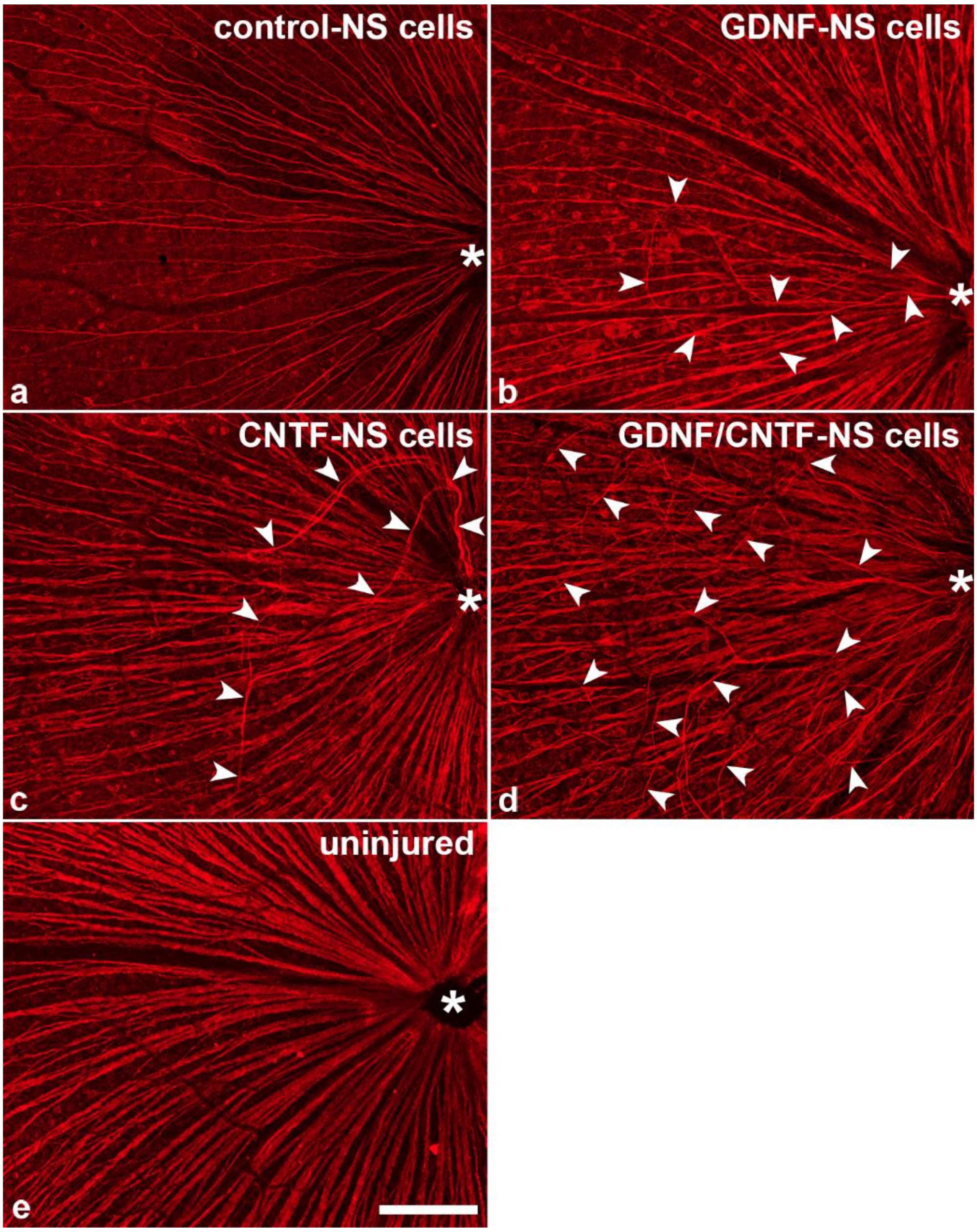


**Figure S4.** Intraretinal growth of retinal ganglion cell axons. ß-tubulin III-positive ganglion cell axons in flat-mounted retinas 8 months after an intraorbital crush and intravitreal transplantations of control-NS (**a**), GDNF-NS (**b**), CNTF-NS (**c**) or GDNF/CNTF-NS cells (**d**). A retina from an uninjured animal is shown for comparison (**e**). All images were taken close to the optic disc (asterisks in **a-e**). Axon fascicles in eyes with grafted GDNF-, CNTF- or GDNF/CNTF-NS cells were significantly thicker than in eyes with grafted control-NS cells. Note the aberrant trajectories of some axon fascicles in GDNF- or CNTF-treated retinas (arrowheads in **b** and **c**, respectively). A pronounced disorganization of the nerve fiber layer was observed in GDNF/CNTF-treated retinas (arrowheads in **d**). Scale bar: 200 µm.

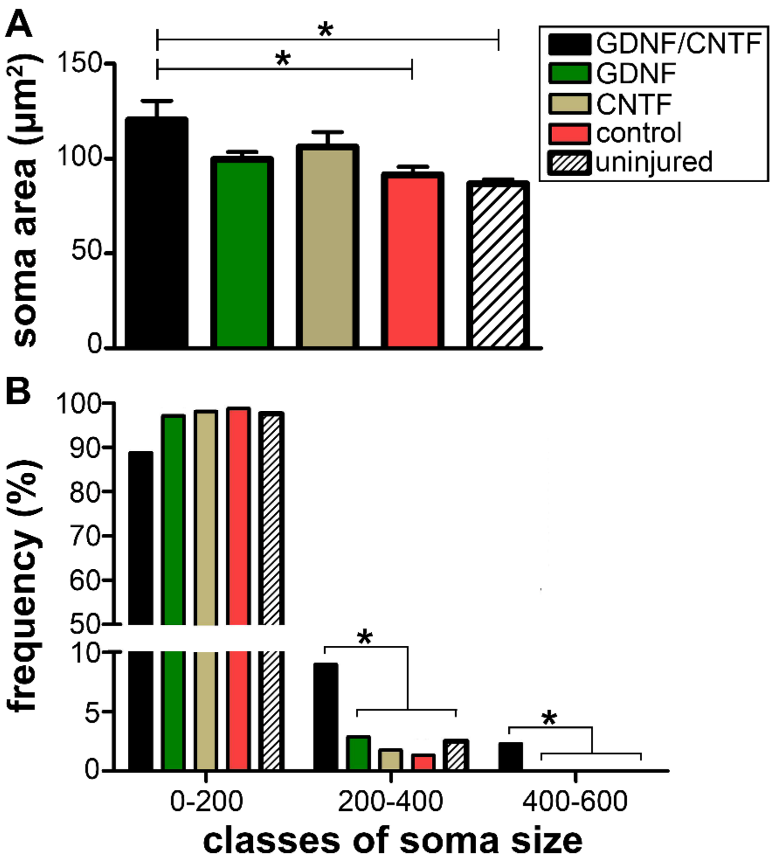


**Figure S5.** Soma size of retinal ganglion cells. (**A**) Eight months after the lesion, the soma size of RGCs was significantly increased in GDNF/CNTF-treated retinas when compared to retinas from control or uninjured animals. Each bar represents the mean (±SEM) from five retinas. *, $p<0.05$ according to the one-way ANOVA followed by a Bonferroni post-hoc test. (**B**) Analyses of soma size classes revealed that GDNF/CNTF-treated retinas contained more large-sized RGCs with a soma size of 200-400 $\mu m^2$ and 400-600 $\mu m^2$ when compared to all other experimental groups and uninjured mice. *, $p<0.05$ according to the Chi-squared test.
